# Stochastic optimization of broadband reflecting photonic structures

**DOI:** 10.1038/s41598-018-19613-6

**Published:** 2018-01-19

**Authors:** D. Estrada-Wiese, E. A. del Río-Chanona, J. A. del Río

**Affiliations:** 1 Instituto de Investigación en Ciencias Básicas y Aplicadas, Universidad Autónoma del Estado de Morelos, Av. Universidad No. 1001 Col. Chamilpa, 62209 Cuernavaca Morelos, Mexico; 2 Centre for Process Systems Engineering, Department of Chemical Engineering, Imperial College, SW7 2AZ London, UK; 3 Instituto de Energías Renovables, Universidad Nacional Autónoma de México, Privada Xochicalco S/N, 62580 Temixco Morelos, Mexico

## Abstract

Photonic crystals (PCs) are built to control the propagation of light within their structure. These can be used for an assortment of applications where custom designed devices are of interest. Among them, one-dimensional PCs can be produced to achieve the reflection of specific and broad wavelength ranges. However, their design and fabrication are challenging due to the diversity of periodic arrangement and layer configuration that each different PC needs. In this study, we present a framework to design high reflecting PCs for any desired wavelength range. Our method combines three stochastic optimization algorithms (Random Search, Particle Swarm Optimization and Simulated Annealing) along with a reduced space-search methodology to obtain a custom and optimized PC configuration. The optimization procedure is evaluated through theoretical reflectance spectra calculated by using the Equispaced Thickness Method, which improves the simulations due to the consideration of incoherent light transmission. We prove the viability of our procedure by fabricating different reflecting PCs made of porous silicon and obtain good agreement between experiment and theory using a merit function. With this methodology, diverse reflecting PCs can be designed for any applications and fabricated with different materials.

## Introduction

The unique optical features of confining light or controlling the propagation of electromagnetic waves in photonic crystals (PCs) enable many important device applications. Waveguides^1^, Bragg-mirrors^2^, surface plasmonic structures^3^, nanocavities and nano-lasers^4,5^ are some examples of optical devices that can be fabricated using PCs. These structures have in common that they are formed of periodically arranged dielectric materials, and their functionality depends highly on the distribution of those materials. Photons entering a PC interact with its periodic dielectric constant and are consequently organized into photonic bands. Analogously to electrons in a crystal, their propagation will be limited by photonic band gaps (PBGs) where transmission states are forbidden. Hence, different designed PCs constrain the propagation of the photons (electromagnetic waves) within the structure depending on their periodic arrangements.

The simplest PCs are one-dimensional and are composed of alternating high and low refractive index layers. These structures can be designed to interact with the electromagnetic waves in such a manner that photonic mirrors (also called Bragg-reflectors) are formed, where “perfect” reflection for specific wavelengths can be achieved. Broadband mirrors can also be engineered if the reflection of a broader wavelength range is needed. These broader mirrors can be achieved by overlaying different Bragg reflectors where each one reflects around a specific central wavelength, resulting in a wider PBG. However, choosing the proper central wavelengths and hence the layer configuration for these Bragg reflectors to superpose adequately is no simple task; furthermore designing such reflecting structures may involve complicated and unpractical methods.

In the context of this work, previous reports have been focused on the PBG enlargement based on staggered structures^6,7^ or chirped gratings^8–10^, amongst others. Commonly, predetermined structure parameters such as layer number, the amount of Bragg reflectors or layer thickness functions constraint to experimental parameters, limit the versatility of these methods. In this paper, we present a practical method to custom design and then evaluate the fabricated broadband PC mirrors. This procedure can be applied to different dielectric materials and easily modified to design other PC structures such as filters for example.

Generally selecting the optimal number of layers and determining their thickness, related to the reflectance wavelength range, represents the main difficulty when designing broadband reflecting PCs. For practical purposes it is pursued that only the wavelength range for the desired application needs to be defined; also, the size of the multilayer correlates directly to the fabrication time which is sought to be the lowest possible. Hence, in this work, we present a stochastic optimization method that finds the best PC layer configuration to build any reflecting structure within the selected wavelength range. This procedure is based on evaluating all possible PCs using their theoretical reflectance spectra and choosing the most reflective PC as the optimized structure. Here we used the equispaced thickness method^11^ to simulate the PCs reflectance spectra considering the effect of incoherent light transmission through the layers based on the transfer matrix formalism. With this approach, we are able to model more realistic multilayered structures which may present imperfections or interface roughness of the fabricated structures leading to scattering effects which need to be considered.

Given the above requirements, to design high quality PCs, topology optimization must be conducted. When addressing topology optimization, previous studies have focused on the use of gradient-based algorithms through sensitivity analysis^12–15^. Moreover, it should be noted that in previous reports, the optimization was done over linearized PCs for short bandwidths. The optimization of PCs for short bandwidths does not present the extra complexity of being multimodal (*i.e*. having many local minima), the current study explores a much wider range of bandwidths which presents severe nonlinearities resulting in multimodality of the solution space (*i.e*. many local minima). While gradient-based algorithms are efficient in many cases, they present two main drawbacks for the current research. First, gradient-based algorithms are of a local search nature, which is ideal when confronted with convex optimization tasks; however, the problem presented in this study is highly nonlinear and nonconvex. Second, the combination of derivative calculations and derivative evaluations is computationally intensive for the current application. Furthermore given that the simulations of the reflectance spectra consist of functions with sharp gradients (*e.g*. exponential, cosines, sines), first order approximations might be ineffective and can lead to convergence problems from the line search^16^ framework. Therefore, the topological optimization in this research is performed through a stochastic optimization algorithm particularly tailored for the current application.

In order to test the performance of this optimization procedure, one dimensional PCs structures need to be fabricated. Production of one dimensional PCs can be achieved using several experimental techniques limited to the fabrication of multilayers with large refractive index contrast. Porous silicon (P-Si) is a nanostructured material widely used to fabricate one-dimensional PCs due to the tunability of its porosity and simple fabrication procedure. In this study, we prove the use and viability of our optimization method by fabricating three different reflecting PCs and using a merit function we measure the differences between theory and experiment finding excellent agreement.

The paper is composed as follows. In section 1 we present a mode to calculate the central wavelength distribution of the Bragg mirrors which form a broadband mirror. In section 2 the stochastic optimization method developed for the search of an optimized reflecting PC structure is shown. The results of the simulations and the fabrication of the optimized broadband mirrors are shown in section 3; where the agreement between theory and simulations is also discussed and evaluated using a merit function. In section 4 the concluding remarks are presented followed by the Methods section where the calculations of the theoretical reflectance spectra, the optimization algorithm and the fabrication of P-Si are described.

## Central wavelength distribution

Designing a broadband photonic mirror requires an adequate superposition of individual Bragg reflectors (named submirrors) that conform it. Each submirror (*n*_*s*_ = 1, …, *n*_*i*_, …, *f* ) creates a PBG that forbids EM transmission around the central wavelength (Λ(*n*_*s*_)) it is designed for. In this manner, to reflect over a wide wavelength range it is necessary to find an appropriate distribution of Λ(*n*_*s*_) for the submirrors to superpose in an optimized way. In recent work, we proposed a method based on the Padé approximant to find each central wavelength^17^, where we defined its distribution as:

1 $${\rm{\Lambda }}({n}_{s})=\frac{a+{a}_{1}{n}_{s}}{1+{b}_{1}{n}_{s}},$$

here *a*, *a*_1_ and *b*_1_ are unknown coefficients; to find them we propose the following system of equations:

2 $${\rm{\Lambda }}(1)=\frac{a+{a}_{1}1}{1+{b}_{1}1},\,{\rm{\Lambda }}({n}_{i})=\frac{a+{a}_{1}{n}_{i}}{1+{b}_{1}{n}_{i}},\,{\rm{\Lambda }}(f)=\frac{a+{a}_{1}\,f}{1+{b}_{1}\,f}\mathrm{.}$$

The central wavelength of the first Λ(1) and the last Λ(*f*) submirrors determine the reflected wavelength range that is required and consequently define the PBG of the broadband mirror. To solve the system of equations 2 we need to know which submirror is the intermediate *n*_*i*_ and its corresponding central wavelength Λ(*n*_*i*_). These two parameters represent two of the optimization parameters to find the best PC structure to construct a broadband mirror.

The alternating layers that form each submirror need to satisfy the quarter wavelength condition which relates the layer thickness *d*_*j*_ and its refractive index *η*_*j*_ to the central wavelength as *η*_*j*_*d*_*j*_ = Λ(*n*_*s*_)/4. The number of layers that form the PC structure is also important to optimize because it defines the width of the entire multilayer and correlates directly to the fabrication time. In general, if the number of submirrors is increased the reflectance spectrum of the broadband mirror presents fewer oscillations, and the quality of the PC reflector is enhanced. However, if the structure is formed of too many layers, the fabrication time increases substantially achieving an ineffective manufacturing process. For example, when fabricating PC mirrors with P-Si and the etching time is large (>2 hours) a porosity gradient in the layers is formed due to the change of the electrolyte concentration. This effect generates a widening of the PBG towards lower frequencies, and although we have boarded this issue previously in a qualitatively way^17^, we will not address this matter in this work. Nevertheless, we need an adequate number of submirrors to design and fabricate optimized PC mirrors. Thus we finally consider three optimization parameters in our stochastic optimization method: Λ(*n*_*i*_), *n*_*i*_ and *n*_*s*_. Each set of these parameters represent the coordinates of particles in a three-dimensional space (the solution space) for all possible PCs at a defined wavelength range. Here we search for the particle with the most reflective spectrum by using the stochastic optimization method presented below. At the end, we use the coordinates of this particle to fabricate the optimized PC mirror with P-Si and prove the usability of our procedure.

## Stochastic optimization Method

The optimization task performed in this work is to find the best possible PC layer configuration with a maximum reflectivity in the selected wavelength range. To this aim a stochastic optimization algorithm is used to explore the solution space of all possible PCs, with respect to the performance criteria presented in equation 5 (obtained in the Methods section): $RP={\int }_{{\rm{\Lambda }}(1)}^{{\rm{\Lambda }}(f)}Rd\lambda$. The highest value of *RP* relates to the maximum reflectivity of the PC mirror that we want to optimize through an algorithm. The algorithm developed for this purpose should possess good exploration and exploitation capabilities to seek an optimal solution efficiently. In simple words, the exploration process refers to the ability of an optimization algorithm to seek for the global optimum in the solution space of an unknown optimization problem, while the exploitation process refers to its ability of applying the knowledge of previous and current solutions to look for better ones. However, the processes of the exploration and the exploitation have conflicting goals, the two must be well balanced to achieve a good optimization^16^. These processes can be better understood when a gradient-based approach and a random search algorithm are compared. On one hand, a gradient-based approach has great exploitation capabilities, as it very well exploits the information of previous and current solutions, however, it easily gets trapped by local minima. On the other hand, a random search algorithm can find a global solution if space is searched widely enough, but its complete lack of exploitation makes it an inefficient algorithm in general.

To address the aforementioned challenges and obtain good optimization performance, three stochastic optimization methods are combined to obtain a hybrid algorithm. This hybrid algorithm uses a number *N* of particles with coordinates (Λ(*n*_*i*_), *n*_*i*_, *n*_*s*_) as its agent and searches the solution space through a combined form of: a) Random Search (RS), b) Particle Swarm Optimization (PSO) and c) Simulated Annealing (SA). Also, when the algorithm determines that there are no good solutions outside a specific domain, the search space is reduced to focus efforts in areas which are more likely to have a high-quality solution. The algorithm, as well as the reasons behind this combination, are described in the Methods section.

## Results

**Figure 1:**
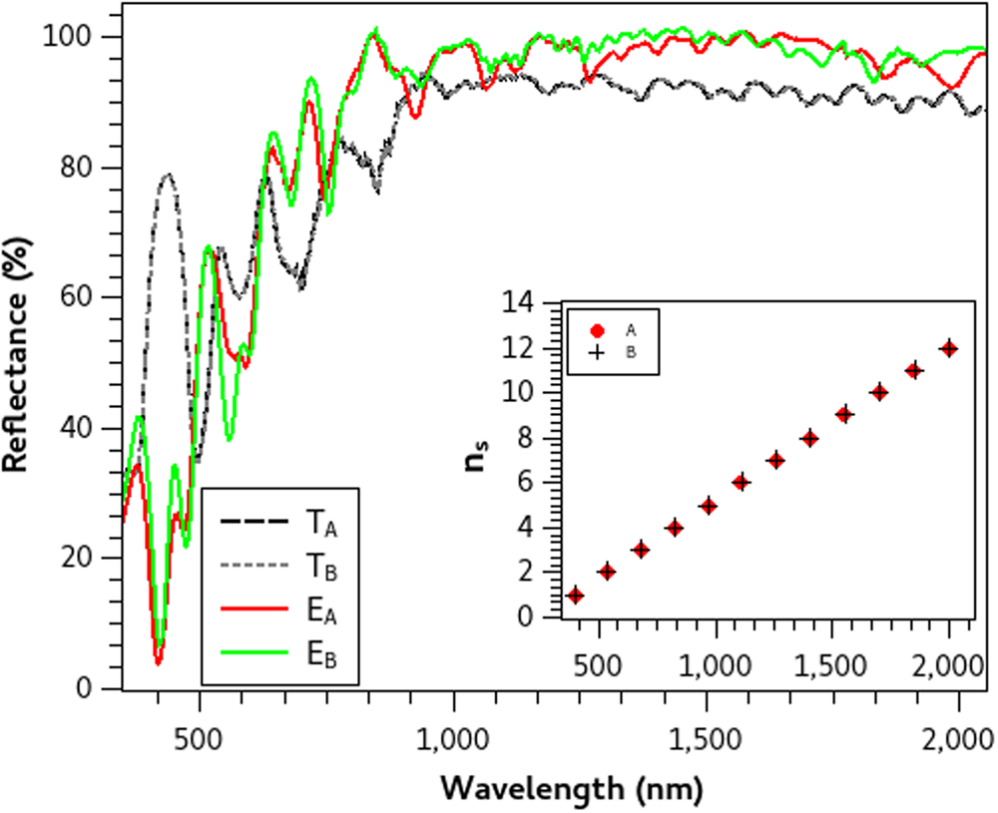
Theoretical and Experimental Reflectance spectra of mirrors *M*_1_.Theoretical (*T*_*A*_, *T*_*B*_) and Experimental (*E*_*A*_ and *E*_*B*_) reflectance spectra of broadband mirrors A and B designed to reflect from 400 to 2000 nm. Their central wavelength distribution Λ(*n*_*s*_) (inset figure) are obtained from the optimized parameters.

**Figure 2:**
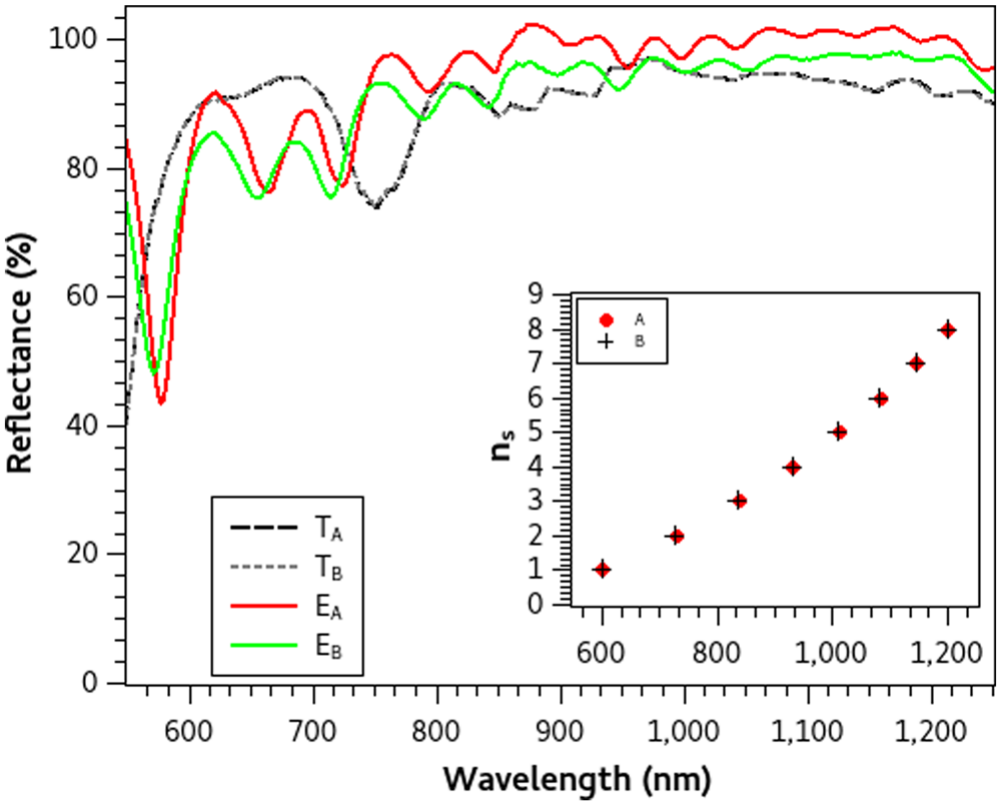
Theoretical and Experimental Reflectance spectra of mirrors *M*_2_.Theoretical (*T*_*A*_, *T*_*B*_) and Experimental (*E*_*A*_ and *E*_*B*_) reflectance spectra of broadband mirrors A and B designed to reflect from 600 to 1200 nm. Their central wavelength distribution Λ(*n*_*s*_) (inset figure) are obtained from the optimized parameters.

**Figure 3:**
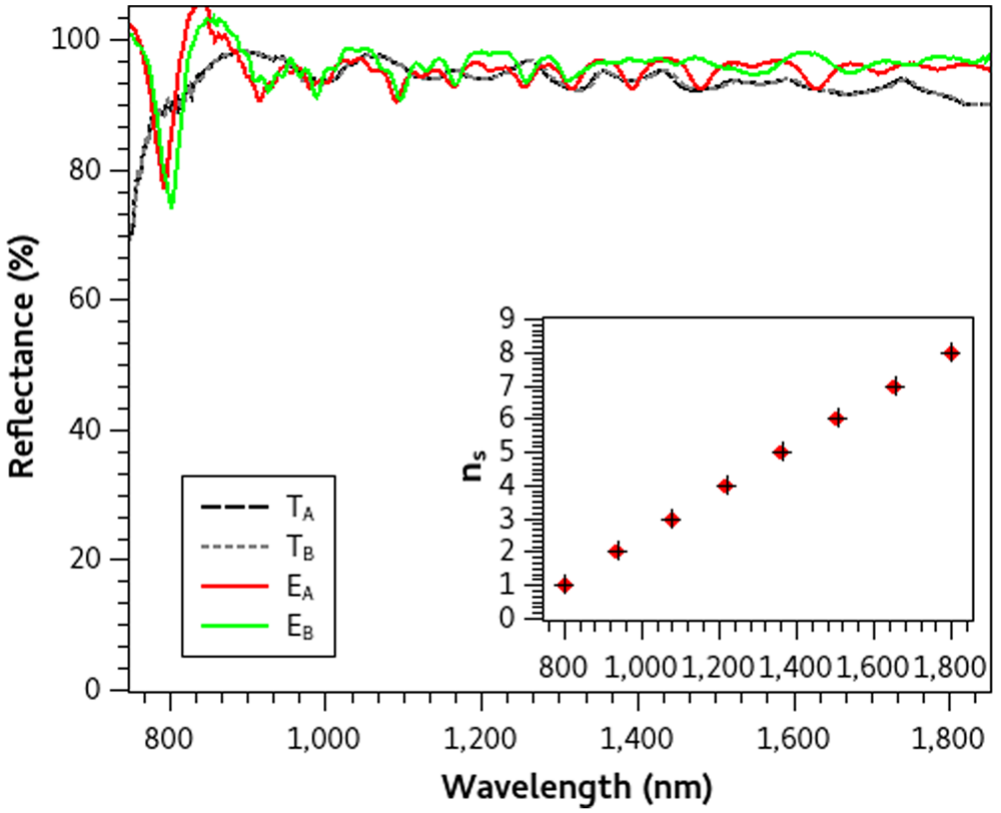
Theoretical and Experimental Reflectance spectra of mirrors *M*_3_.Theoretical (*T*_*A*_, *T*_*B*_) and Experimental (*E*_*A*_ and *E*_*B*_) reflectance spectra of broadband mirrors A and B designed to reflect from 800 to 1800 nm. Their central wavelength distribution Λ(*n*_*s*_) (inset figure) are obtained from the optimized parameters.

In this study, we present three different optimized reflecting PCs each one designed to inhibit electromagnetic wave transmission over diverse and wide wavelength ranges. The first mirror *M*_1_ is designed to reflect from Λ(1) = 400 to Λ(*f*) = 2000 nm, the second *M*_2_ from Λ(1) = 600 to Λ(*f*) = 1200 nm and the third *M*_3_ from Λ(1) = 800 to Λ(*f*) = 1800 nm. We restrict our study only for *p* = 5, which seems to be an adequate number of periods and hence used this value for all the calculations. We ran the optimization algorithm twice for each wavelength range and obtained slight different optimized particles (each one with different coordinates) for all mirrors but with very similar *RP* values between every pair (*A* and *B*). Thus the optimization algorithm finds optimal PC configurations in every search, even though they have small differences in their central wavelength distribution. The optimized parameters of the three pairs of mirrors are summarized in Table 1 where the similarities can be compared. Given these values we solved equation 1 and found the central wavelength distribution Λ(*n*_*s*_) of each PC configuration. Insets in Figs 1–3 are the Λ(*n*_*s*_) of mirrors A and B respectively where no significant differences can be observed. From the quarter wavelength condition, we were able to calculate the layer thicknesses of the submirrors which form the PC structures and afterward we used this information to finally fabricate the mirrors.

**Table 1:** Optimization parameters (Λ(*n*_*i*_), *n*_*i*_, *n*_*s*_) of the reflecting PCs that we designed in this study which were obtained from the stochastic optimization method. Their reflectance performance criteria *RP* are also enlisted.

PC	Λ(*ni*) (nm)	*n*_*i*_	*n*_*s*_	*RP*
*M*_1*A*_	966	5	12	136’506
*M*_1*B*_	1114	5	12	136’507
*M*_2*A*_	838	3	8	55’022
*M*_2*B*_	929	4	8	55’021
*M*_3*A*_	1076	3	8	94’521
*M*_3*B*_	1508	6	8	94’519

Once the layer thicknesses of each PC configuration were defined, we fabricated all the aforementioned mirrors with P-Si following the fabrication procedure described in the Methods section. The experimental and theoretical spectra of each pair of mirrors are compared in Figs 1–3 where no differences between the theoretical spectra are observed, but slight disagreements in the experimental counterparts are found. In general, there is a very good agreement between simulations and experiments, but we need a quantitative measure to calculate the differences which we present in the next section.

## Discussion

The theoretical reflectance spectra of the PC mirrors represent the performance criteria of each optimized structure. In practice these calculated spectra are attempted to be reproduced with the experiments; hence we validated the accuracy of the fabricated mirrors using a merit function as a quantitative quality measure between theory and experiment. Here we define the merit function as

3 $$N=\sqrt{\frac{{\int }_{{\rm{\Lambda }}(1)}^{{\rm{\Lambda }}(f)}{(T(\lambda )-E(\lambda ))}^{2}d\lambda }{{({\int }_{{\rm{\Lambda }}(1)}^{{\rm{\Lambda }}(f)}T(\lambda )d\lambda )}^{2}}},$$

where *T*(*λ*) and *E*(*λ*) are the simulated and experimental reflectance spectra, respectively. The best agreement between experiments and theory requires *N* to be small. The optimization method that we present in this report is based on evaluating the most reflective theoretical spectra of each PC structure, furthermore, we use this method and find an adequate experimental agreement of theoretical and experimental spectra. We emphasize that to have quantitative comparison we calculated the merit function for all the mirrors that we fabricated within this study and find good agreement for all the samples. In Table 2 we resume the obtained values of *N* which show differences of order 10^−5^ between the mirrors A and B of *M*_1_ and *M*_3_ and differences of order 10^−4^ between the mirrors *M*_2_. Thus, these results show the viability of our optimized methodology to design and fabricate broadband high reflective PC structures.

**Table 2:** Values of the merit function *N* and fabrication time (*t*_*f*_) of the reflecting PCs presented in this report.

PC	*N* (×10^−3^)	*t*_*f*_ (h)
*M*_1*A*_	4.41	1.78
*M*_1*B*_	4.36	1.78
*M*_2*A*_	3.83	0.93
*M*_2*B*_	3.23	0.93
*M*_3*A*_	1.11	1.30
*M*_3*B*_	1.20	1.30

Besides fabrication and reproduction quality, the time diminution of the process is important to be considered. In previous work, we have reported P-Si mirror structures which are as reflective as the optimized mirrors we present here, but their multilayer sizes are much larger because of their large submirror number (*n*_*s*_ = 20)^17^. These mirrors take 3.5 h at least to be fabricated which double the time used to produce the optimized structures presented in this paper (see Table 2). Therefore by using the stochastic optimization method to design and then fabricate the PC mirrors we have improved the efficiency of the custom mirror making process.

We stress that this optimization method which balances exploration and exploitation and combined with the solution space reduction can also be employed to optimize 2D or 3D PCs efficiently. The only necessary adaptation to optimize 2D and 3D structures would be to define an adequate form of constructing the possible PCs along with customized performance criteria for the desired application, as we did with the Padé central wavelength distribution for the 1D case.

## Concluding Remarks

In this research, we have developed a practical design procedure to find the most reflective broadband PC within a desired wavelength range. First, we consider the alternating layers of each submirror formed with two materials of different refractive indexes, which can be wavelength dependent. Second, to form the broadband PC, we vary three design parameters: the central wavelength of each submirror, the distribution of these central wavelengths, and the number of Bragg mirrors to cover the complete wavelength range of reflection. To find an optimized high reflective PC, we combined three stochastic optimization methods and obtained a hybrid algorithm which furthermore includes a space reduction strategy to search for the optimized PC. Also, we validate this procedure by fabricating several PCs applying the optimization method, and we measure the agreement between theory and experiment by means of a merit function. The results show good agreement and also prove efficient fabrication times which add to the improvement of the optimization method to design reflecting PCs. We want to stress tree main contributions of this paper: first, the novel optimization algorithm constructed in this work can be used to optimize other highly nonlinear and nonconvex systems; second the validation against experimental performance of the optimized theoretical PCs; and third the results are not only valid for PC made with porous silicon multilayers. The last implies that with this procedure any reflecting PC structure can be designed for different applications and fabricated with either material adequate for PC manufacturing.

## Methods

### Simulation of theoretical reflectance spectra

**Figure 4:**
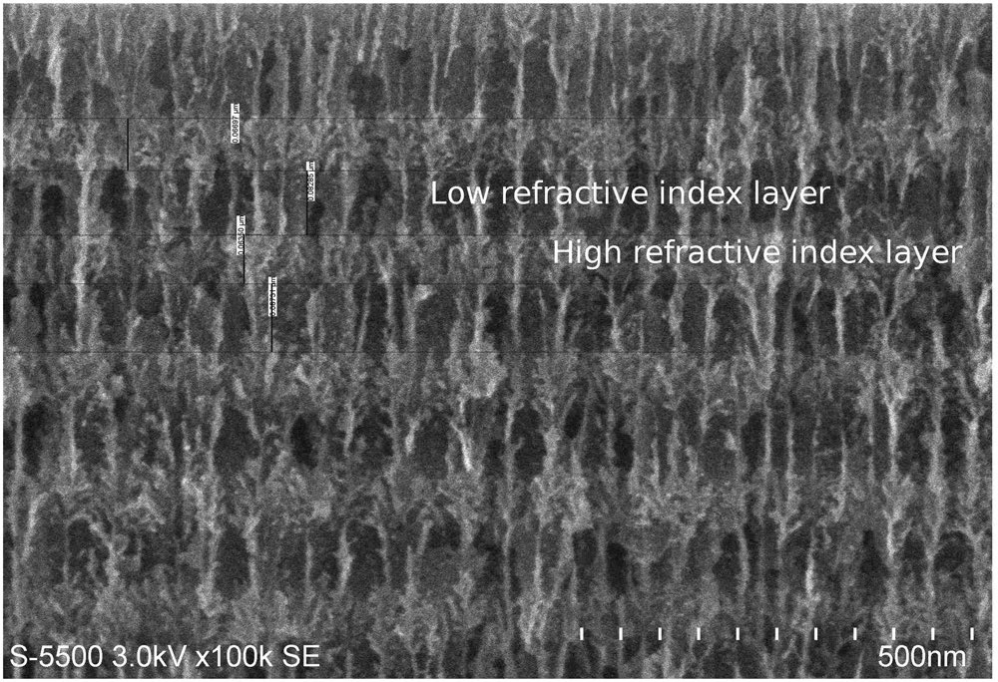
SEM image of mirror. SEM image of a porous silicon photonic mirror where the high and low refractive index layers are shown.

One of the most common tools to calculate multilayer structures scattering spectra (transmission, reflection, and absorption) is the transfer-matrix method (TM)^18^. However, the form of the conventional TM assumes coherent light propagation through a plane and homogeneous layers which may result in unrealistic spectra calculations. In practice, layers formed of P-Si, for example, have nonuniform interfaces presenting rough surfaces as shown in the SEM image of Fig. 4. The interaction of light with these imperfections lead to destructive interference effects that can be introduced in the simulations by considering incoherent layers in the system. The equispaced thickness method (ETM) proposes a form to incorporate these effects by including equidistant variations in the thickness of the layers^11^. Using the TM formalism the transmission or reflectance spectra for each variation is calculated and then averaged over the equispaced thicknesses.

In this study we use the ETM to simulate the reflectance spectra of each desired PC structure; moreover, these spectra represent the evaluation function of our stochastic optimization method. Within the TM theory, the electromagnetic field propagation in the structure formed of *l* layers is represented by the matrix

$$[\begin{array}{c}{E}_{I}\\ {H}_{I}\end{array}]={\bf{M}}[\begin{array}{c}{E}_{(l+\mathrm{1)}}\\ {H}_{(l+\mathrm{1)}}\end{array}],$$

where *E*_*I*_, *H*_*I*_, *E*_(*l* + 1)_, *H*_(*l* + 1)_ are the electrical and magnetic fields in the first and the last interface, respectively. The transfer matrix ***M*** is defined by the product of the characteristic matrices ${{\bf{M}}}_{{d}_{j}}$ of each layer from *j* = 1, 2, … *l* as

$${\bf{M}}={{\bf{M}}}_{{d}_{1}}{{\bf{M}}}_{{d}_{2}}\cdots {{\bf{M}}}_{{d}_{l}}\mathrm{.}$$

For a transverse electric field at normal incidence, the transfer matrix is a function of the complex refractive index *η*_*j*_, the thickness *d*_*j*_ of the layer *j* and the wavelength *λ* of the electric field

4 $${{\bf{M}}}_{{d}_{j}}\equiv {{\bf{M}}}_{{d}_{j}}({\eta }_{j},\lambda )=(\begin{array}{cc}\cos \,(\frac{2\pi {\eta }_{j}{d}_{j}}{\lambda }) & i\,\sin (\frac{2\pi {\eta }_{j}{d}_{j}}{\lambda })/{Y}_{j}\\ {Y}_{j}i\,\sin (\frac{2\pi {\eta }_{j}{d}_{j}}{\lambda }) & \cos (\frac{2\pi {\eta }_{j}{d}_{j}}{\lambda })\end{array})\mathrm{.}$$

Here *Y*_*j*_ is determined by ${Y}_{j}=\sqrt{\frac{{\varepsilon }_{0}}{{\mu }_{0}}}{\eta }_{j}$, where *ε*_0_ and *μ*_0_ are the vacuum permittivity and the permeability.

Because the PC mirrors are formed of *n*_*s*_ number of submirrors (*n*_*s*_ = 1, ..., *f* ), with *p* number of periods composed of layers of high (*H*) and low(*L*) refractive indexes, the total transfer matrix for this structure is constructed by multiplying iteratively the submirror matrices

$${\bf{M}}={({{\bf{M}}}_{{d}_{H}\mathrm{(1)}}{{\bf{M}}}_{{d}_{L}\mathrm{(1)}})}^{p}{({{\bf{M}}}_{{d}_{H}\mathrm{(2)}}{{\bf{M}}}_{{d}_{L}\mathrm{(2)}})}^{p}\cdots {({{\bf{M}}}_{{d}_{H}(f)}{{\bf{M}}}_{{d}_{L}(f)})}^{p}\mathrm{.}$$

The reflection coefficient can be written in terms of the elements *m*_*ij*_ of **M**, here *Y*_*a*_ stands for the incident media (air):

$$r=\frac{{Y}_{a}{m}_{11}+{Y}_{a}{Y}_{(l+\mathrm{1)}}{m}_{12}-{m}_{21}-{Y}_{(l+\mathrm{1)}}{m}_{22}}{{Y}_{a}{m}_{11}+{Y}_{a}{Y}_{(l+\mathrm{1)}}{m}_{12}+{m}_{21}+{Y}_{(l+\mathrm{1)}}{m}_{22}},$$

from here the reflectance is obtained as

$$R=|r{|}^{2}\mathrm{.}$$

The reflectance spectrum of a PC multilayer is simulated by calculating *R* for all wavelengths within the defined range. To incorporate the equispaced thicknesses in the previous description we redefine the thickness of each submirror layer as *d*_*k*_(*n*_*s*_) = Λ(*n*_*s*_)/4*η*_*k*_(*n*_*s*_) + *δ* where *k* = *H*, *L* and *δ* represents the variance of the thickness due to the surface roughness or inhomogeneities which cause incoherent light propagation. In this study, we define *δ* as a function of the measured layer rugosity *S* (see the Porous silicon fabrication Section for detailed explanation of the determination of *S* of P-Si) in the form

$$\delta =S\cdot i/{X}_{d}$$

where *i* takes values from 1 to *X*_*d*_, the number of equidistant values. For each given value of *i*, we calculate the corresponding reflectance spectrum of the multilayer which forms the PC. Then we have a collection of *X*_*d*_ reflectance spectra and we average them over the equispaced thickness values to finally obtain the simulated spectrum of the PC mirror. In this study, we used *X*_*d*_ = 5 after finding that a larger number did not significantly change the simulations.

Following on we perform a numerical integration of the averaged reflectance spectrum to determine its reflectance performance criteria (*RP*) that is needed for the stochastic optimization method as the evaluation function. For the numerical integration we first apply a linear interpolation to the spectrum and then integrate over the wavelength range of the PC mirror:

5 $$RP={\int }_{{\rm{\Lambda }}(1)}^{{\rm{\Lambda }}(f)}Rd\lambda $$

In this manner we have defined the mathematical problem which is nonlinear and presents nonconvex features. Therefore we require an optimization method able to deal with these characteristics. The main objective is to evaluate *RP* for each different PC structure in the parametric three dimensional space comparing and classifying the optimization algorithm described below to find the most reflective PC which corresponds to the PC with the highest *RP* value.

### Optimization algorithm

The algorithm proposed in this research uses three main phases: Initialization, Evaluation and Classification and Space Exploration.

1. Initialization
  In this phase, *N* particles are randomly placed in the solution space of the optimization problem.
2. *Evaluation and Classification*
  The value of *RP* for each different PC structure in the solution space is compared and classified accordingly to the optimization algorithm described below to find the most reflective PC which corresponds to the PC with the highest *RP* value. In this phase, the performance criteria of each particle is evaluated, after which particles are classified into three groups, and the group they are assigned to will determine the search strategy they will follow.
  *N*_*SA*_ particles with the highest evaluated function will follow an SA search strategy for the following *n* iterations.
  *N*_*RS*_ particles with the lowest evaluated function will follow an RS search strategy for the following *n* iterations.
  The remaining *N*_*PSO*_ particles will follow a PSO search strategy for the following *n* iterations.
3. *Space Exploration*
  The above classification works in such a way that the position of each particle is better exploited depending on the information it can supply to the swarm as a whole.
  The *N*_*SA*_ particles should be a small number of particles which are assumed to be near high-quality solutions and therefore should intensively explore their neighborhood. To this end, SA, an efficient stochastic optimization algorithm of a local nature is used. The particular implementation in this work follows from^19^.
  A similar reasoning is applied to the *N*_*PSO*_ particles. In the solution space, these particles are positioned such that they might not be close to high-quality solutions, however by using the knowledge of other particles in the swarm they can search for better solution areas which have not yet been found by other particles. PSO is mainly designed for this purpose, as it is inspired in a flock of birds collectively exchanging information and foraging for food, likely to move closer to an optimum of a fitness function. The implementation in this work follows from^20^.
  Finally, the *N*_*RS*_ particles are those assumed to be relatively far from any high-quality solution, and hence exploring the neighborhood around them would not bring benefit to the swarm. The *No Free Lunch Theorem*^21^ establishes a theoretical proof that any optimization algorithm is in average as good as the Random Search strategy, and that the advantage of specialized algorithms is in using and obtaining knowledge about the problem at every iteration. Given that particles that are too far away from optimal solutions can hardly provide useful information, then particles classified as *N*_*RS*_ are reinitialized randomly.
4. *Repeat*
  After *n* iterations the algorithm either returns to the *Evaluation and Classification* phase or terminates.
5. *Space reduction*
  During the algorithm, the best position of each particle in the current cycle is recorded. After *N*_*cycle*_ iterations, the search space is reduced to the smallest hypercube that contains the best position of all particles in the current cycle, this terminates the cycle. Subsequently, the algorithm returns to the *Initialization* phase.

**Figure 5:**
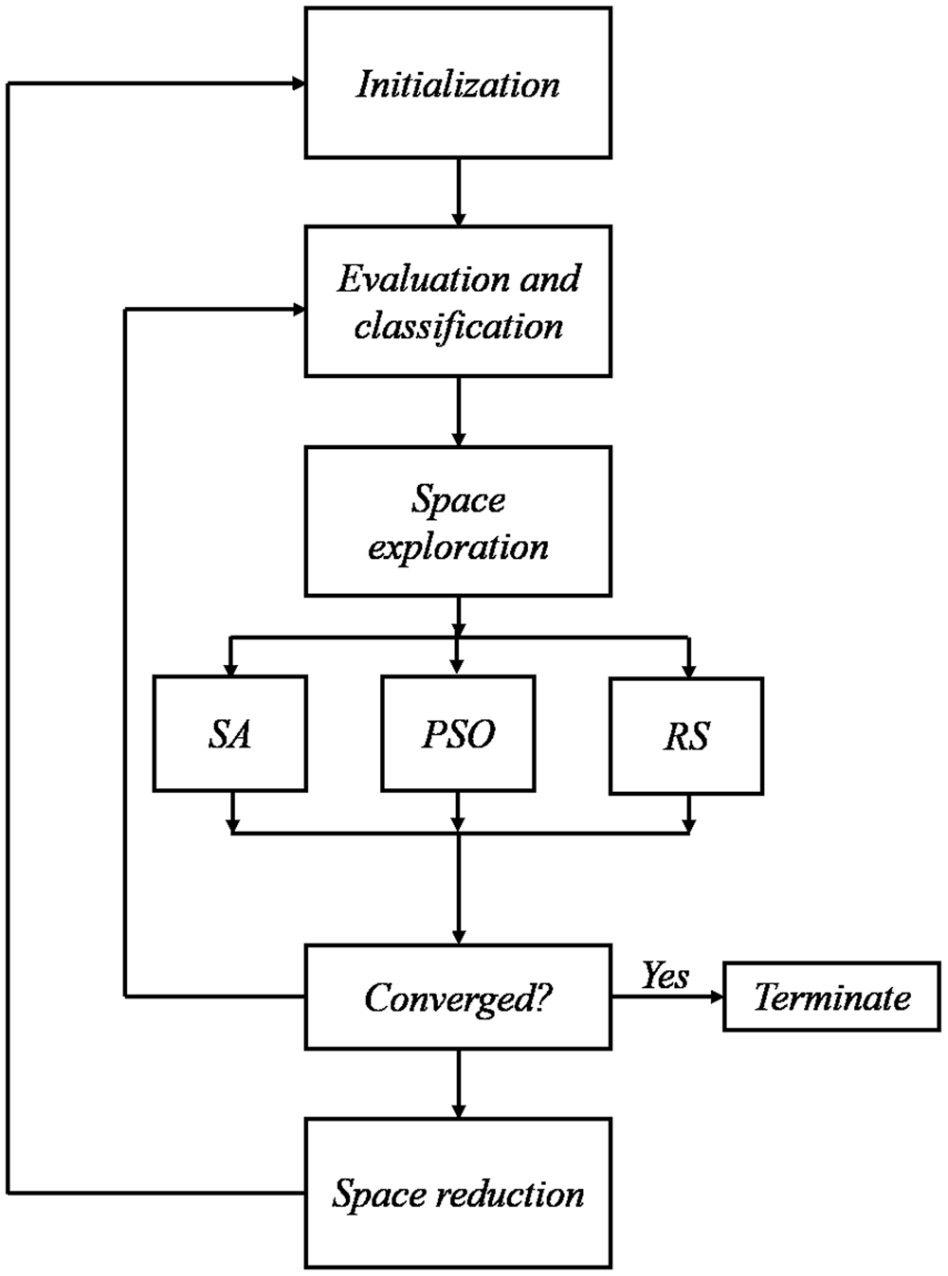
Diagramm of optimization algorithm. Diagrammatic representation of stochastic optimization algorithm.

In this way, given that RS, PSO, and SA are in ascending order of exploration and in descending order of exploitation a balance in the exploration-exploitation paradigm can be achieved. Furthermore, the space reduction allows the algorithm to focus efforts in the areas that are most likely to have a high quality (or possibly the global) optimum. Figure 5 shows a schematic representation of the optimization algorithm.

It is demonstrated in the Results Section that this algorithm is highly efficient in finding the best solution for the design of PCs.

### Porous silicon PC fabrication

Porous silicon (P-Si) PCs were prepared by electrochemical anodization of highly boron-doped p^+^-type (100) crystalline silicon wafers with resistivity <0.005 Ω ⋅ *cm* in a hydrofluoric acid solution composed of ethanol, HF and glycerin in a volume ratio of 7:3:1 (70 ml of ethanol, 30 ml of HF and 10 ml of glycerol). We fabricated high and low refractive index layers by alternating the current density from 3.0 *mA*/*cm*^2^ to 40.0 *mA*/*cm*^2^ respectively. The corresponding refractive index values, needed for the design of the PC structure, were obtained by using the Bruggeman approximation as reported in^22^. Using etching rates of *v*_*a*_ = 14.49 *nm*/*s* and *v*_*b*_ = 1.72 *nm*/*s* we calculated the time at which each current density needed to be applied to form the desired thickness of the layers. Also, to avoid HF concentration decrease which causes a porosity gradient in the formed layers, we implemented 1 second long pauses to the etching time. After electrochemical etching, the samples were rinsed in ethanol for 10 minutes and dried under a nitrogen stream. We subsequently oxidized the samples for stabilization of the P-Si at 300 °C during 15 minutes.

The morphology of P-Si varies with porosity, and as shown in the SEM image 4 the high and low porosity layers (low and high refractive index respectively) fabricated in this study resemble a coral-like structure. The surfaces between layers are not smooth and present rugosity *S*. One way to determine *S* is to measure the maximal variations in the layer thicknesses which can be obtained from SEM imaging. Here we measured the layer thicknesses of our samples using a Hitachi S5500 electron microscope and averaged over the measurements finding *S* = 7.6 ± 0.1 nm.

Experimental reflectance spectra were measured using an UV-VIS-IR Spectrophotometer (Shimadzu UV1601) in three different places of each sample to asess homogeneity. The average of these measurements (*R*_*exp*_) are normalized with regard of a standard aluminium reflectance spectrum (*R*_*Al*_) using the relation: *E*_*norm*_ = (*R*_*exp*_ ⋅ *R*_*Al*_)/100. These normalized reflectance spectra *E*_*norm*_ are presented in Figs 1 to 3 as the experimental reflectance spectra (*E*_*A*_ and *E*_*B*_) of each fabricated PC mirror.

## References

[1] Li J, White TP, O’Faolain L, Gomez-Iglesias A, Krauss TF (2008). Systematic design of flat band slow light in photonic crystal waveguides. Opt. Express.

[2] Agarwal V, del Rí JA (2006). Filters, Mirrors and Microcavities from Porous Silicon. Int. J. Modern Phys. B.

[3] Barnes WL, Dereux A, Ebbesen TW (2003). Surface Plasmon subwavelength optics. Nature.

[4] Noda S, Fujita M, Asano T (2007). Spontaneous-emission control by photonic crystals and nanocavities. Nature Photon..

[5] Nozaki K, Kita S, Baba T (2007). Room temperature continuous wave operation and controlled spontaneous emission in ultrasmall photonic crystal nanolaser. Opt. Express.

[6] Kumara V, Anisb M, Singha KS, Singhb G (2011). Large range of omni-directional reflection in 1D photonic crystal heterostructures. Optik.

[7] Zhanga H, Liua S, Konga X, Biana B, Zhaoa H (2012). Properties of omnidirectional photonic band gap in one-dimensional staggered plasma photonic crystals. Opt. Commun..

[8] Mouldi A, Kanzari M (2012). Design of an omnidirectional mirror using one dimensional photonic crystal with graded geometric layers thicknesses. Optik.

[9] Ariza-Flores AD, Gaggero-Sager LM, Agarwal V (2012). White metal-like omnidirectional mirror from porous silicon dielectric multilayers. Appl. Phys. Lett..

[10] Archuleta-Garcia R, Moctezuma-Enriquez D, Manzanares-Martinez J (2010). Enlargement of Photonic Band Gap in Porous Silicon Dielectric Mirrors. J. of Electromagn. Waves and Appl..

[11] Kang K (2016). A Simple Numerical Modeling of the Effect of the Incoherent Thick Substrate in Thin-Film Solar Cells Based on the Equispaced Thickness Method. IEEE Photonics J..

[12] Jensen JS (2007). Topology optimization of dynamics problems with Padé approximants. Int. J. Numer. Meth. Engng.

[13] Dahl J, Jensen JS, Sigmund O (2008). Topology optimization for transient wave propagation problems in one dimension. Struct. Multidisc. Optim..

[14] Smajic J, Hafner C, Erni D (2004). Optimization of photonic crystal structures. J. Opt. Soc. Am. A.

[15] Jensen JS, Sigmund O (2011). Topology optimization for nano-photonics. Laser Photonics Rev..

[16] Li G, Niu P, Xiao X (2012). Development and investigation of efficient artificial bee colony algorithm for numerical function optimization. Appl Soft Comput..

[17] Estrada-Wiese D (2015). Staggered Padé wavelength distribution for multi-Bragg photonic mirrors. Sol. Energ. Mater. Sol. Cells.

[18] Hecht, E. Óptica (Adelphi University 2002), Chap.9.

[19] Xiang Y, Gong XG (2000). Efficiency of generalized simulated annealing. Phys. Rev. E..

[20] Zambrano-Bigiarini, M., Clerc, M., & Rojas, R. Standard Particle Swarm Optimisation 2011 at CEC-2013: A baseline for future PSO improvements. In 2013 IEEE Congress on Evolutionary Computation (pp. 2337–2344). IEEE. 10.1109/CEC.2013.6557848 (2013).

[21] Wolpert DH, Macready WG (1997). No free lunch theorems for optimization. IEEE Trans. Evol. Comput..

[22] Estrada-Wiese, D. & del Río, J. A. Refractive index evaluation of porous silicon using Bragg reflectors. Preprint at: https://arxiv.org/abs/1711.03117v1 (2017).

